# Case-Fatality Risk of Norovirus, England, 2022–2025 

**DOI:** 10.3201/eid3208.260091

**Published:** 2026-08

**Authors:** Maria L. Tang, Amy Douglas, Cristina Celma, Roberto Vivancos, Gauri Godbole, Thomas Ward, Jonathon Mellor

**Affiliations:** UK Health Security Agency, London, UK (M.L. Tang, A. Douglas, C. Celma, R. Vivancos, G. Godbole, T. Ward, J. Mellor); University of Oxford, Oxford, UK (A. Douglas); National Institute for Health and Care Research, Norwich, UK (R. Vivancos); University of Warwick, Coventry, UK (R. Vivancos).

**Keywords:** norovirus, viruses, enteric infections, viral infections, genotype, case-fatality risk, mortality, severity, epidemiology, surveillance data linkage, England

## Abstract

Norovirus incidence increased in England during 2022–2025, when GII.17 replaced GII.4 as the dominant genotype. By using nationally linked norovirus testing and fatality data, we found age and care setting, but not genotype, were associated with case-fatality risk. Increased incidence might reflect changes in transmissibility or population immunity.

Norovirus is a highly contagious virus that causes diarrhea and vomiting. Although symptoms from norovirus are not inherently severe, norovirus places substantial pressure on healthcare systems, particularly in closed settings where transmission rates are high. Older adults and hospitalized patients are disproportionately affected and are at greater risk of severe outcomes and complications (*1*,*2*).

Noroviruses comprise 10 genogroups (GI–GX) and 48 genotypes (*3*), and cross-immunity is limited (*4*). During the past decade, the GII.4 genotype predominated globally (*5*), with semiregular strain replacement (*6*). During the 2023–24 winter season, GII.17 reemerged and replaced GII.4 as the dominant genotype in multiple countries (*7*). In the 2024–25 season, record-high laboratory reports of norovirus were recorded in England, driven by the co-circulation of GII.17 and GII.4 genotypes (*8*). The increase in reported norovirus cases might reflect greater disease severity, increased transmissibility, or reduced population immunity to GII.17, although distinguishing among those explanations is challenging (*9*), particularly with aggregate data (*10*).

Whereas previous studies have investigated norovirus severity across genotypes (Appendix), we directly compared the severity of disease caused by GII.4 and GII.17 genotypes during the recent shift in genotype dominance. We estimated genotype-specific case-fatality risk to compare the relative severity of norovirus strains by linking individual-level laboratory, genotyping, hospitalization, and death data from England during the 2022–2025 seasons. 

## The Study

In this retrospective cohort study, we included persons in England with a positive norovirus test, linking routine individual-level laboratory test results to death and hospitalization records. We included positive test results from the UK Health Security Agency’s modular open laboratory information system (MOLIS) (*11*) for genotyped specimens and the second generation surveillance service (SGSS) (*11*), which lacks genotyping information but captures a larger, more representative set of tests. We obtained death registrations from the Office of National Statistics through July 23, 2025 (*12*). 

**Figure 1:**
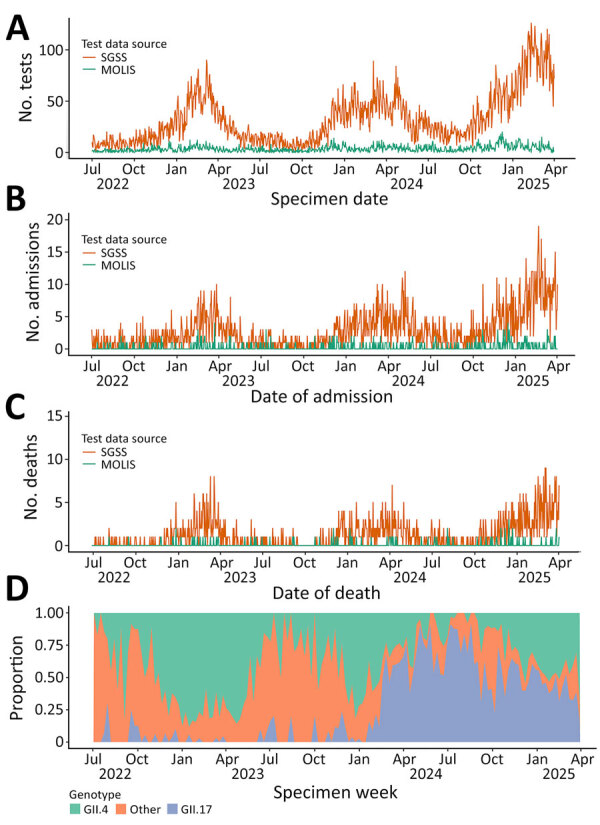
Number and proportion of positive norovirus test results and linked severe outcomes from a study of norovirus case-fatality risk in England during the 2022–23 and 2024–25 seasons. A) Number of de-duplicated positive norovirus test results. B) Number of hospital admissions within 14 days after a linked positive norovirus test result. C) Number of deaths occurring within 14 days of a linked positive norovirus test result. D) Proportion of norovirus genotypes among positive cases identified through the MOLIS. MOLIS, Modular Open Laboratory Information System; SGSS, Second Generation Surveillance Service.

We linked records by using National Health Service number (unique patient identifier) and relevant dates (Figure 1; Appendix). By using MOLIS and SGSS data, we attributed deaths to norovirus infection episodes by linking each death to any positive norovirus test within the preceding 14 days (Appendix). From those data, we generated 3 linked datasets: MOLIS-deaths, SGSS-deaths, and their intersection, MOLIS-SGSS-deaths. We also analogously linked hospitalizations to positive tests (Appendix).

We used piece-wise exponential additive mixed models to estimate hazard ratios and risk of death after a positive norovirus test (case-fatality risk) (Appendix). We specified the model structure on the basis of the causal structure (Appendix) and domain expert opinion, adjusting for age, genotype, healthcare level, and laboratory region. In addition, we used stratifications for specimen date quarter, capturing potential temporal changes in virulence, healthcare-seeking behavior, or hospital pressures (Appendix). We fitted separate models to the MOLIS-deaths, the SGSS-deaths, and the MOLIS-SGSS-deaths datasets.

For 2022–2025, we observed seasonal norovirus case and hospitalization waves in England (Figure 1). GII.4 dominated the 2022–23 season, but GII.17 became the predominant genotype mid-2023 through mid-2024, continuing into the 2024–25 season. We observed a higher proportion of positive test results and severe outcomes in the 2024–25 seasons, which correlated with the reemergence of GII.4 in early 2025.

Positive test results occurred primarily in older adults and infants; the MOLIS dataset showed a greater representation of infants than the SGSS dataset (Appendix Figure 4). Deaths occurred predominantly among older adults. The unadjusted case-fatality ratio was highest among older adults and lowest among infants (Appendix Tables 2, 3). The unadjusted case-fatality ratio was higher for persons infected with GII.4 than among those infected with GII.17, and lower among persons infected with other genotypes (Appendix Tables 2, 4). During the peak of the 2024–25 season, the average case-fatality risk in the MOLIS-deaths cohort (median age 64, modal region North West) for all-cause death within 14 days of a positive norovirus test result was 3.11% (95% CI 1.29%–7.60%) for genotype GII.4 and 1.74% (95% CI 0.65%–4.67%) for GII.17 (Appendix Table 5). 

**Figure 2:**
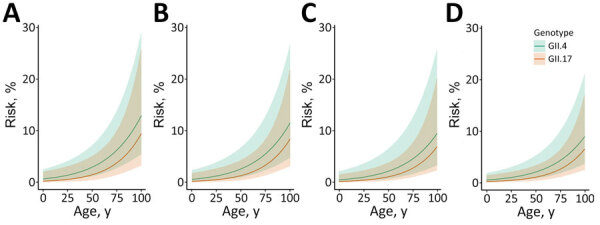
Predicted 14-day case-fatality risk after a positive norovirus test result in England during the 2022–23 and 2024–25 seasons. A) Calendar quarter beginning January 2023. B) Calendar quarter beginning January 2024. C) Calendar quarter beginning April 2024. D) Calendar quarter beginning January 2025. Data represent model-predicted probabilities of death within 14 days after a positive norovirus test result; shading indicates 95% CIs, estimated by using the positive test results from the Modular Open Laboratory Information System. Predictions are shown for the modal region (North West), stratified by norovirus genotype and time period. Time-period facets were chosen to reflect periods of peak activity in each season; 2 periods are shown for the 2023–24 season to reflect periods of GII.4 or GII.17 genotype dominance.

Case-fatality risk for the 2022–23 and 2023–24 seasons was similar. Case-fatality risk by age increased monotonically, similarly between the 2 genotypes (Figure 2). We did not observe a significant difference between estimated case-fatality risk for GII.4 and GII.17 cases (Appendix Figure 6), but risk varied between sequencing laboratory regions. We observed similar results by using 28- or 60-day thresholds to associate deaths with a positive norovirus test (Appendix).

**Figure 3:**
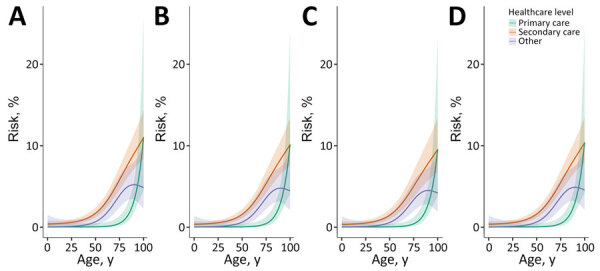
Predicted 14-day case fatality risk after a positive norovirus test result in England during the 2022–23 and 2024–25 seasons. A) Calendar quarter beginning January 2023. B) Calendar quarter beginning January 2024. C) Calendar quarter beginning April 2024. D) Calendar quarter beginning January 2025. Data represent model-predicted probabilities of death within 14 days after a positive norovirus test result; shading indicates 95% CIs, estimated by using the positive test results from the Second Generation Surveillance Service. Predictions are for tests taken in the modal region (North West), stratified by healthcare level and time period. Time-period facets were chosen to reflect periods of peak activity in each season; 2 periods are shown for the 2023–24 season to reflect periods of GII.4 or GII.17 genotype dominance.

The all-cause death case-fatality risk within 14 days of a positive norovirus test was 4.64% (95% CI 3.68%–5.85%) in secondary care and 0.42% (95% CI 0.15%–1.16%) in primary care. During the 2024–25 seasonal peak, case-fatality risk increased monotonically by age for all healthcare levels (Figure 3). All-cause risk was significantly higher for cases identified in secondary care than in primary care for most ages (hazard ratio at median age 0.089 [95% CI 0.033–0.239]) (Appendix Figure 7). The case-fatality risk was significantly higher for cases identified in secondary care compared with other healthcare settings for older adults only; we found no statistical difference for other ages (hazard ratio at median age 0.67 [95% CI 0.44–1.01]). Those patterns were consistent but stronger and evident across a wider age range for 28- or 60-day death thresholds (Appendix). We did find a significant difference in risk between persons tested in different regions. Genotype and healthcare level (primary vs. secondary) effects modeled from the smaller MOLIS-SGSS-deaths data were not significant (Appendix).

## Conclusions

By estimating genotype-specific case-fatality risk, we found no evidence that genotype GII.17 was associated with a higher risk of death than genotype GII.4 in England. The recent increase in norovirus cases is likely driven by changes in transmissibility, population immunity, or testing dynamics than increased genotype-specific severity.

Although our linkage and modeling of individual-level data enabled estimation of genotype-specific fatality risk, several limitations should be considered (Appendix). We estimated all-cause case-fatality risk among norovirus cases, which likely overestimates norovirus-attributable death, particularly in secondary care and compared with other studies (Appendix). Underascertainment of milder cases might further inflate case-fatality risk estimates, and residual confounding, including underlying conditions, remains despite individual-level adjustment.

The absence of a detectable difference in case-fatality risk between genotypes GII.4 and GII.17 suggests the 2024–25 wave of severe outcomes was unlikely driven by greater severity of GII.17. Instead, increased transmissibility or reduced population immunity likely contributed to an increase in severe norovirus cases, consistent with limited population exposure to genotype GII.17 after prolonged GII.4 dominance (*4*,*7*). Our results have important implications for the ongoing development of norovirus mRNA vaccines (*13*) because strain selection and composition are carefully considered to maximize effectiveness. Further work is needed to understand if norovirus genotype or reduced population immunity contributed to the increase in severe norovirus cases (Appendix). After the completion of this study, norovirus became a notifiable condition in England, which might improve future predictions of case-fatality risk (Appendix). Taken together, our results suggest that, although GII.17 is not more severe than GII.4, an increase in reported severe norovirus cases demands public health actions including prioritizing hygiene and cleaning in community and healthcare settings.

Appendix Additional information on case-fatality risk of norovirus, England, 2022–2025.
