# Supplementary material for: Case-Fatality Risk of Norovirus, England, 2022–2025
Source: Emerg Infect Dis. 2026 Aug;32(8):1336–40. doi: 10.3201/eid3208.260091 (PMC13426853; doi:10.3201/eid3208.260091)
Supplement: Appendix — Additional information on case-fatality risk of norovirus, England, 2022–2025. [file 26-0091-Techapp-s1.pdf]

Article DOI: https://doi.org/10.3201/eid3208.260091

EID cannot ensure accessibility for supplementary materials supplied by authors. Readers who have difficulty accessing supplementary content should contact the authors for assistance.

Case-Fatality Risk of Norovirus, England, 2022–2025

Appendix

Section 1: Previous work on norovirus severity

Previous studies have compared severity and transmissibility between norovirus genotypes, but none yet for the recent GII.17 strain. Seasons dominated by GII.4 have shown higher hospitalization and mortality rates than years with dominant non-GII.4 genotypes (*1*,*2*). However, during the emergence of a new GII.4 variant in England in 2002/03, the relative mortality risk among elderly individuals was lower than in other seasons (*3*). Overall, the study estimated 80 norovirus-attributable deaths per year in individuals aged 65 years or older in England. Other studies have estimated norovirus severity but not by genotypes. Among US veterans, norovirus was associated with a slight increase in hospitalization risk and a larger increase in mortality risk that decreased over time (*4*).

Case-fatality risk (CFR), the probability that a reported case results in death, is a key measure of disease severity. CFR in studies can vary widely due to differences in age, comorbidities and setting (e.g. hospital, residential care) (*5*, *6*), and whether all-cause or norovirus-attributable deaths are used. A review of 52 international studies found high heterogeneity in CFR, with an overall model-based CFR of 0.025% (95% CI: 0.003%, 0.195%) (*7*). CFR from seven studies of residents in long-term care facilities ranged from 0.3% to 1.6% (*8*). Most previous work relies on aggregate data or data from specific subpopulations, which makes national-level inferences more difficult.

Section 2: Data

Genotyping data

Genotyping data in England came from the UK Health Security Agency's (UKHSA) Modular Open Laboratory Information System (MOLIS) (*9*). The Enteric Virus Unit (EVU) genotype a subset of norovirus-positive specimens for surveillance. Positivity is confirmed in all specimens upon arrival in EVU (*10*). Genotype assignment of positive specimens is performed by sequencing as described elsewhere (*11*). Most norovirus-positive specimens were referred by the seven regional laboratories, covering the nine English regions (with single laboratories serving both the West and East Midlands, and London and the East of England). Guidance at the time requested laboratories sent up to three norovirus-positive specimens associated with any one hospital or community outbreak, and up to five further randomly selected non-outbreak specimens each week, prioritizing specimens with the highest viral load based on PCR cycle threshold values. Fewer specimens came from local National Health Service (NHS) laboratories which were encouraged to refer specimens for genotyping during winter 2024/25 in response to the rise in GII.17, selecting up to two specimens per week with the highest viral load.

SGSS data

Diagnostic laboratories in England have a statutory duty to report positive detection of notifiable organisms and do this electronically, through UKHSA's Second Generation Surveillance Service (SGSS) (*9*). We extracted positive norovirus test results from England from SGSS, which captures a larger set of tests more representative of overall burden but lacks genotyping. A range of norovirus detection tests are used in diagnostics laboratories, information that is not available for this study. SGSS is one of the three systems used by UKHSA to monitor epidemiological trends for norovirus (*12*). SGSS also contains more demographic and contextual information, including patient residential geography and the healthcare level that requested the test, such as general practice (GP), hospital inpatient, hospital outpatient and accident & emergency (A&E).

De-duplication of test result data

De-duplication of MOLIS test results retained each person's first positive test result and any subsequent positives occurring at least 21 days after the previous retained positive; however, positive results within 21 days with a different genotype from the previous result were also

retained. Specimens that could not be genotyped due to insufficient viral load or technical limitations were classified as “Unknown” genotype and only retained if they represented a person’s sole positive test.

We select a 21-day episode threshold to balance the competing priorities of infection event classification. Long episode durations risk grouping multiple infection episodes together, whereas short durations could separate a single episode into multiple. We select 21 days as an interval greater, but not substantially larger, than the average viral shedding duration of norovirus patients which is often 14 days or greater (*12*,*13*, *14*, *15*), with longer durations particularly in the elderly, who consist of the majority of our cases (Appendix Figure 4).

As with the MOLIS data, de-duplication of SGSS data retained the first positive test result and any subsequent positives occurring at least 21 days after the previous retained positive.

Hospitalization data

Hospitalization data came from the Secondary Uses Service Admitted Patient Care (SUS APC) dataset, with both hospital spell and episode records for admissions to NHS hospitals (*16*). Hospital spells and episodes were extracted up to admission dates of 4 August 2025. Hospital spells represent a continuous hospital stay from admission to discharge and may consist of multiple episodes of care, each under a different consultant. Hospital episodes carry diagnostic codes from the International statistical classification of diseases, 10th revision (ICD-10) (*17*). We look at hospital episodes with the diagnosis code A08.1, which represents acute gastroenteropathy due to norovirus and other small round viruses, and so may not constitute a confirmed diagnosis of norovirus, however more specific diagnosis codes were not available.

Hospital admissions (at the spell or episode level) were linked to the nearest positive test result in time, as tests may be taken before or after hospitalization. For MOLIS cases with a linked hospitalization, tests are mostly taken after admission (Appendix Figure 5).

Data linkage

Test results, deaths and hospitalizations were linked using NHS number (unique patient identifier) and relevant dates (Appendix Figure 1).

It is difficult to attribute deaths to norovirus infections, as most deaths occur in the elderly, who may have multiple comorbidities. We could not use cause of death for attribution,

as most deaths were recorded to be due to non-specific causes such as old age or frailty. Instead, we used a time-based threshold as a proxy, defining norovirus-associated mortality as death occurring within 14 days after a positive norovirus test result. This constitutes all-cause mortality, which may overestimate deaths attributable to norovirus infection.

Deaths were separately linked to infection episodes from the MOLIS and SGSS data by linking each death to a positive result occurring within the 14-day window prior to death. When multiple test results met this criterion, the most recent test result was selected, assuming more recent infection episodes are more attributable to deaths. Sensitivity of results on the 14-day death threshold is detailed in the supplementary material. In MOLIS cases with a linked death, most deaths are seen within 60 days (Appendix Figure 5, Appendix Table 1).

To link MOLIS and SGSS test results, each MOLIS test result was linked to the closest SGSS test result within a 21-day window either side, according to specimen dates. Some MOLIS test results from regional laboratories did not have corresponding SGSS entries. Most linked MOLIS-SGSS pairs shared identical specimen dates; for those that did not, the earliest specimen date of the two was used in the following analysis.

Section 3: Causal structure

Causal relationships contributing to death of norovirus cases, whether attributable to norovirus or not, are shown in Figure 2. Observed data include age and some healthcare characteristics (level of healthcare, region), but other patient demographics, healthcare characteristics (e.g. specialism and size) and comorbidities that affect risk of death are unobserved. Healthcare system pressure can also increase mortality risk due to resource constraints – although unobserved, temporal variation can be partially adjusted for by including calendar time in our model.

Not all individuals with norovirus have the same probability of being tested, introducing selection bias. Observed severity among the cohort of ascertained cases is therefore not representative of severity across all infections. Supplementary Figure 3 shows the causal relationships contributing to someone with norovirus getting tested. Patient demographics, comorbidities and residential setting (e.g. care home) influence underlying risk of severe disease, but also healthcare-seeking behavior and healthcare access, which contribute to probability of

testing. Healthcare characteristics and system pressure further affect these and influence other factors like testing capacity and de-prioritization of routine norovirus testing during outbreaks of other pathogens (*18*).

Section 4: Case-fatality model

Piece-wise exponential additive mixed models (PAMMs) flexibly model time-to-event data with non-linear and time-varying effects using generalized additive mixed model methods (Bender, Groll, & Scheipl, 2018). PAMMs were fit to time from the specimen date of a positive norovirus test until death or censoring. Test results were followed up until the earliest of death, a number of days after the specimen date equal to the deaths threshold, the specimen date of the next test result of that individual, or the data cutoff date. In the main results, the deaths threshold is 14 days. In the supplementary, we also consider 28 and 60 days as sensitivity analysis. When follow-up ended on or before the specimen date, follow-up time was set to 1 day. Follow-up was split into daily intervals from specimen date for MOLIS-deaths and MOLIS-SGSS-deaths, reflecting the daily resolution of specimen and death dates. For SGSS-deaths models, two-day intervals were used to reduce computational load.

Covariates were included for genotype, healthcare level and lab region. Genotype was grouped as "GII.4", "GII.17", "Other" and "Unknown". Healthcare level was grouped as "Primary care" (including only GP), "Secondary care", "Other" and "Unknown". Unknown values of categorical values (genotype and healthcare level) were retained so patients with missing variables could still contribute to global effects of their non-missing variables. In the MOLIS-deaths data, 368/3542 records had "unknown" genotype (Table 1). In the SGSS-deaths data, 3,729/32,427 records had "unknown" healthcare levels (Appendix Table 3). MOLIS-deaths models used MOLIS lab regions (7 NHS regions), whereas SGSS-deaths models used SGSS lab regions (9 ONS regions). MOLIS-SGSS-deaths models used broader grouped regions ("North", "Midlands", "South") due to smaller sample sizes. Records with missing age values were removed from the model data (1 or 2 records, Appendix Figure 1).

Based on the DAG (Figure 2) and subject matter expert opinion elicitation, we specified the following hazard functions $h_i(t)$ for person-record-episode i at time t (time since specimen date):

MOLIS-deaths:

$\log(h_i(t)) = \log(h_{0,\text{quarter}_i}(t)) + \beta_{\text{genotype}_i} + f(\text{age}_i) + f_{\text{genotype}_i}(\text{age}_i) + \beta_{\text{lab region}_\text{i}} + \omega_{\text{person}_i}.$

MOLIS-SGSS-deaths:

$\log(h_i(t)) = \log(h_{0,\text{quarter}_i}(t)) + \beta_{\text{genotype}_i} + f(\text{age}_i) + f_{\text{genotype}_i}(\text{age}_i) + \beta_{\text{lab region}_\text{i}} + \beta_{\text{healthcare level}_\text{i}} + f_{\text{healthcare level}_i}(\text{age}_i) + \omega_{\text{person}_i}.$

SGSS-deaths:

$\log(h_i(t)) = \log(h_{0,\text{quarter}_i}(t)) + f(\text{age}_i) + \beta_{\text{lab region}_\text{i}} + f_{\text{healthcare level}_i}(\text{age}_i) + \beta_{\text{healthcare level}_\text{i}} + \omega_{\text{person}_i}.$

Here, $\log(h_{0,\text{quarter}_i}(t))$ are stratum-specific log baseline hazards for specimen date quarter. This captures potential temporal changes in strain virulence, healthcare-seeking behaviour, healthcare access, or hospital pressures that could influence severity. Specimen date quarter is used due to the sparse data in some months in the MOLIS-deaths and MOLIS-SGSS data, and also used for SGSS-deaths models for consistency. β are fixed effects, $f(\text{age}_i)$ are global splines fit to age, $f_{\text{genotype}_i}(\text{age}_i)$ and $f_{\text{healthcare level}_i}(\text{age}_i)$ are age splines specific to genotypes and healthcare levels, and ω_{person_i} are person-level random effects to account for multiple record-episodes per individual. We include genotype and healthcare-level-specific age effects to allow age-related severity patterns to differ across groups.

Section 5: Case-fatality risk and ratio terminology

In this paper, we use the term "case-fatality risk" for the estimated model-based probability of death among cases and "case-fatality ratio" for crude unmodelled deaths-to-cases ratios. Our use of "case-fatality risk" represents an estimand from our piecewise-exponential additive mixed model. From our model, we estimate survival probabilities, and the reported quantity is the model-based probability of death by a specified time among individuals meeting the case definition, calculated as one minus the survival probability. We therefore use "case-fatality risk" to reflect that the estimand is a time-specific conditional probability of death among cases. This usage is consistent with Kelly and Cowling, who argue that case fatality estimates a

conditional probability and may therefore be considered a risk (*19*), and with outbreak-methods literature defining the case-fatality risk as the probability that a case dies from the infection (*20*). In contrast, we reserve the term "case-fatality ratio" crude deaths divided by reported cases.

Further, case-fatality estimates are not the same as infection-fatality estimates. Case-fatality estimates depend on the case definition (in this study, an individual with a recorded positive norovirus test result), and so our estimates are affected by case ascertainment and biased by healthcare-seeking and testing behaviors. As test-positive cases only represent a subset of all infections (and likely skewed towards more severe infections), our case-fatality estimates should not be interpreted as population-wide mortality rates.

Section 6: MOLIS-SGSS-deaths

We can also link the MOLIS-deaths dataset to SGSS test results to get the subset of MOLIS-SGSS-deaths, which includes additional information on the healthcare level that requested the test. In the smaller MOLIS-SGSS-deaths dataset, genotypes GII.4 and GII.17 are associated with statistically similar risk of death (Appendix Figure 8), and there is not enough statistical power to detect a difference in effect of cases tested in secondary care from primary care. The age risk profile with large confidence intervals is shown in Appendix Figure 9.

Section 7: Sensitivity to death threshold

In the main results, the threshold for linking deaths to test results is deaths occurring within 14 days. In this section, we re-fit models changing the deaths linkage threshold to within 28 or 60 days.

MOLIS-deaths data

Hazard ratios for genotype and lab region for deaths within 28 and 60 days in the MOLIS-deaths data are shown in Supplementary Figure 10 and Supplementary Figure 11. GII.4 and GII.17 have statistically similar risk of death – this matches the results for deaths within 14 days.

SGSS-deaths data

Hazard ratios for healthcare level and lab region for deaths within 28 and 60 days in the SGSS-deaths data are shown in Appendix Figure 12 and Appendix Figure 13. These give consistent but stronger results as deaths within 14 days. For a 28-day threshold, compared to cases identified in secondary care, those identified in primary care have significantly lower risk of death for all adult ages, and those in other settings have significantly lower risk in all ages except the oldest (Appendix Figure 12). For a 60-day threshold, compared to cases identified in secondary care, risk of death is significantly lower for cases of all ages identified in primary care or other settings (Appendix Figure 13).

MOLIS-SGSS-deaths data

Hazard ratios for genotype, healthcare level and lab region for deaths within 28 or 60 days in the MOLIS-SGSS-deaths data are shown in Supplementary Figures 14 and 15. There is no statistical difference in risk between genotypes GII.4 and GII.17 for both. There is no statistical difference in risk between cases identified in primary or secondary care with a 28-day threshold. For a 60-day threshold, there was limited evidence of a statistically significant difference, restricted to a narrow age range.

Section 8: Strengths and limitations

Use of individual-level mortality records and test results enabled estimation of genotype-specific risk while adjusting for patient and healthcare provider characteristics such as age and location, offering advantages over aggregate CFR approaches. Linkage of test and death data allowed assessment of linkage definitions, showing robustness of hazard ratio results (Appendix Section 6). PAMMs provided flexibility in model structure while accounting for right censoring. However, we were unable to adjust for all confounding factors that can bias our results (*20*), such as patient comorbidities. Sample size constraints also limited power in analyses when adjusting for both genotype and healthcare level.

Because we estimated all-cause mortality among test-positive cases, CFRs are likely inflated relative to norovirus-attributable risk. This may partially explain why the CFR was substantially higher for cases identified in secondary care compared with primary care, and why our CFR is higher than other studies which use diagnosis codes (Appendix Section 1).

Attributing deaths to norovirus is difficult, as severe illness occurs primarily in older adults with comorbidities (*4*, *8*).

Under-ascertainment of cases is also likely to have influenced our estimates. Not all individuals with a norovirus infection seek healthcare, especially those with mild symptoms, and for those who do, not all will be tested for norovirus. Testing practices vary by healthcare setting, disease severity and patient characteristics, with a higher likelihood of testing in secondary care, care homes and among more vulnerable individuals. Further, there are testing biases for which specimens are genotyped, as those with higher viral loads are more likely to be referred (Appendix Section 2). As a result, our case definition of confirmed test-positive individuals represents a subset of all infections and is likely biased toward more severe disease. We therefore expect the estimated case-fatality risk to be lower than the infection-fatality risk, and our CFR estimates should therefore not be interpreted as a population-level mortality risk.

Section 9: Comparison of results with other studies

Direct comparison with CFRs from other studies is challenging due to different cohorts, surveillance capability and death linkage methods. Our estimated cohort CFR is much larger than a model-based CFR of 0.025% (95% CI: 0.003%, 0.195%) from 52 international studies, many of which had small sample sizes and no deaths (*7*). Some studies used norovirus diagnostic codes to attribute deaths, giving a smaller CFR than our all-cause mortality risk. As our results suggest mortality risk greatly increases with age, reported CFRs depend substantially on the cohort's age structure, which may explain regional differences seen in our CFR.

No other studies have directly compared severity between genotypes for the recent dominant GII.17 strain. However, increased activity has been well-documented in multiple countries coinciding with an increase in GII.17 and replacement of GII.4 as the dominant genotype (*21*). Despite this, there has been no clear evidence of a corresponding increase in severity. An indirect comparison in a hospital-based study in Thailand, 2021-2023, found GII.7 samples to have lower viral loads than GII.4, which could indicate lower severity or transmissibility (*22*). Internationally, norovirus reporting is not generally mandatory or routinely linked to clinical outcomes, making severity comparisons between genotypes mainly limited to

outbreak or hospital studies. Historically, GII.4 outbreaks have been linked to higher hospitalization and mortality rates than non-GII.4 outbreaks (*1*, *2*).

Section 10: Future work

This study focused on case-fatality risk, but hospitalization is another important outcome. However, from linked case-hospitalization data, most tests were taken after hospital admission. While tests taken shortly after admission may indicate community-acquired infections which are then hospitalized, the longer delays observed for most cases are more consistent with nosocomial transmission. Hence, this data would not be so appropriate for estimating case-hospitalization risk as most cases were already in hospital prior to contracting norovirus. Nosocomial transmission would disproportionately affect higher risk patients (elderly or comorbid) and hence raise observed CFR values (*23*). Attributing hospitalization to norovirus infection is also difficult when there are multiple comorbidities and diagnosis codes. Using a case definition of individuals with a confirmed positive norovirus test reduces the risk of misdiagnosis but underestimates the true burden of infection. Community studies could improve understanding of severity by estimating infection-hospitalization and infection-fatality risks (*24*).

Future research can investigate how changes in transmissibility, immunity and susceptibility jointly drive genotype replacement events. Further mechanistic transmission modelling of norovirus would help clarify how changes in genotype dominance translate into observed epidemiological outcomes (*25*). Strengthening routine linkage and surveillance of genomic, clinical and demographic data will be crucial for assessing the impact of genotype shifts and informing vaccine development.

Section 11: Implications of future surveillance

After this study's period, norovirus became notifiable in England (6 April 2025), requiring laboratories to report positive results to UKHSA. This will improve completeness of test data and may partially enhance case ascertainment. However, testing will likely remain biased towards more severe cases, inflating CFR estimates of ascertained cases compared to all cases (*20*). Further, the change in reporting could shift testing practice unpredictably. Expanded routine genotyping, including outside health and social care settings, would improve estimates of

genotype-specific severity and allow for adjustment for confounding. Current prioritization of high-viral-load specimens for genotyping is another bias towards severe cases but is operationally necessary to ensure genotyping is possible.

References

1. Desai R, Hembree CD, Handel A, Matthews JE, Dickey BW, McDonald S, et al. Severe outcomes are associated with genogroup 2 genotype 4 norovirus outbreaks: a systematic literature review. Clin Infect Dis. 2012;55:189–93. PubMed https://doi.org/10.1093/cid/cis372

2. Burke RM, Shah MP, Wikswo ME, Barclay L, Kambhampati A, Marsh Z, et al. The norovirus epidemiologic triad: predictors of severe outcomes in US norovirus outbreaks, 2009-2016. J Infect Dis. 2019;219:1364–72. PubMed https://doi.org/10.1093/infdis/jiy569

3. Harris JP, Edmunds WJ, Pebody R, Brown DW, Lopman BA. Deaths from norovirus among the elderly, England and Wales. Emerg Infect Dis. 2008;14:1546–52. PubMed https://doi.org/10.3201/eid1410.080188

4. Cates J, Cardemil CV, Mirza SA, Lopman B, Hall AJ, Holodniy M, et al. Risk of hospitalization and mortality following medically attended norovirus infection-Veterans Health Administration, 2010-2018. Open Forum Infect Dis. 2023;10:ofad556. PubMed https://doi.org/10.1093/ofid/ofad556

5. Mattner F, Sohr D, Heim A, Gastmeier P, Vennema H, Koopmans M. Risk groups for clinical complications of norovirus infections: an outbreak investigation. Clin Microbiol Infect. 2006;12:69–74. PubMed https://doi.org/10.1111/j.1469-0691.2005.01299.x

6. Lopman BA, Adak GK, Reacher MH, Brown DW. Two epidemiologic patterns of norovirus outbreaks: surveillance in England and Wales, 1992-2000. Emerg Infect Dis. 2003;9:71–7. PubMed https://doi.org/10.3201/eid0901.020175

7. Asare EO, Hergott D, Seiler J, Morgan B, Archer H, Wiyeh AB, et al. Case fatality risk of diarrhoeal pathogens: a systematic review and meta-analysis. Int J Epidemiol. 2022;51:1469–80. PubMed https://doi.org/10.1093/ije/dyac098

8. Lindsay L, Wolter J, De Coster I, Van Damme P, Verstraeten T. A decade of norovirus disease risk among older adults in upper-middle and high income countries: a systematic review. BMC Infect Dis. 2015;15:425. PubMed https://doi.org/10.1186/s12879-015-1168-5

9. UK Health Security Agency. Quality and methodology information: national norovirus and rotavirus reports. 2025 Sep 25 [cited 2026 Jan 03]. https://www.gov.uk/government/publications/national-norovirus-and-rotavirus-surveillance-reports-quality-and-methodology-information/quality-and-methodology-information-national-norovirus-and-rotavirus-reports.

10. Kageyama T, Kojima S, Shinohara M, Uchida K, Fukushi S, Hoshino FB, et al. Broadly reactive and highly sensitive assay for Norwalk-like viruses based on real-time quantitative reverse transcription-PCR. J Clin Microbiol. 2003;41:1548–57. PubMed https://doi.org/10.1128/JCM.41.4.1548-1557.2003

11. Gallimore CI, Cheesbrough JS, Lamden K, Bingham C, Gray JJ. Multiple norovirus genotypes characterized from an oyster-associated outbreak of gastroenteritis. Int J Food Microbiol. 2005;103:323–30. PubMed https://doi.org/10.1016/j.ijfoodmicro.2005.02.003

12. Aoki Y, Suto A, Mizuta K, Ahiko T, Osaka K, Matsuzaki Y. Duration of norovirus excretion and the longitudinal course of viral load in norovirus-infected elderly patients. J Hosp Infect. 2010;75:42–6. PubMed https://doi.org/10.1016/j.jhin.2009.12.016

13. Milbrath MO, Spicknall IH, Zelner JL, Moe CL, Eisenberg JN. Heterogeneity in norovirus shedding duration affects community risk. Epidemiol Infect. 2013;141:1572–84. PubMed https://doi.org/10.1017/S0950268813000496

14. Tu ET, Bull RA, Kim MJ, McIver CJ, Heron L, Rawlinson WD, et al. Norovirus excretion in an aged-care setting. J Clin Microbiol. 2008;46:2119–21. PubMed https://doi.org/10.1128/JCM.02198-07

15. Gustavsson L, Nordén R, Westin J, Lindh M, Andersson LM. Slow clearance of norovirus following infection with emerging variants of genotype gii.4 strains. J Clin Microbiol. 2017;55:1533–9. PubMed https://doi.org/10.1128/JCM.00061-17

16. Herbert A, Wijlaars L, Zylbersztejn A, Cromwell D, Hardelid P. Data resource profile: hospital episode statistics admitted patient care (hes apc). Int J Epidemiol. 2017;46:1093–1093i. PubMed https://doi.org/10.1093/ije/dyx015

17. World Health Organization. ICD-10 : international statistical classification of diseases and related health problems : tenth revision, 2nd ed. 2004 [cited 2026 Jan 03]. https://iris.who.int/handle/10665/42980.

18. Ondrikova N, Clough HE, Douglas A, Iturriza-Gomara M, Larkin L, Vivancos R, et al. Differential impact of the COVID-19 pandemic on laboratory reporting of norovirus and Campylobacter in

England: A modelling approach. PLoS One. 2021;16:e0256638. PubMed https://doi.org/10.1371/journal.pone.0256638

19. Kelly H, Cowling BJ. Case fatality: rate, ratio, or risk? Epidemiology. 2013;24:622–3. PubMed https://doi.org/10.1097/EDE.0b013e318296c2b6

20. Lipsitch M, Donnelly CA, Fraser C, Blake IM, Cori A, Dorigatti I, et al. Potential biases in estimating absolute and relative case-fatality risks during outbreaks. PLoS Negl Trop Dis. 2015;9:e0003846. PubMed https://doi.org/10.1371/journal.pntd.0003846

21. Chhabra P, Wong S, Niendorf S, Lederer I, Vennema H, Faber M, et al. Increased circulation of GII.17 noroviruses, six European countries and the United States, 2023 to 2024. Euro Surveill. 2024;29:2400625. PubMed https://doi.org/10.2807/1560-7917.ES.2024.29.39.2400625

22. Kittigul L, Pairoh T, Rupprom K, Thongpanich Y, Siri S. Emergence of norovirus GII.17[P16] in adult patients with acute gastroenteritis in Thailand during 2021-2023. PLoS One. 2025;20:e0337513. PubMed https://doi.org/10.1371/journal.pone.0337513

23. Lopman BA, Reacher MH, Vipond IB, Sarangi J, Brown DW. Clinical manifestation of norovirus gastroenteritis in health care settings. Clin Infect Dis. 2004;39:318–24. PubMed https://doi.org/10.1086/421948

24. Inns T, Harris J, Vivancos R, Iturriza-Gomara M, O'Brien S. Community-based surveillance of norovirus disease: a systematic review. BMC Infect Dis. 2017;17:657. PubMed https://doi.org/10.1186/s12879-017-2758-1

25. Vesga JF, Douglas A, Celma C, Knock ES, Baguelin M, Edmunds WJ. The transmission dynamics of norovirus in England: a genotype-specific modelling study. Epidemics. 2025;53:100875. PubMed https://doi.org/10.1016/j.epidem.2025.100875

Appendix Table 1. Summary of de-duplicated positive test results from MOLIS and linked hospital spell admissions, hospital episodes with norovirus diagnosis (ICD-10 code A081 as a primary or secondary code) or death, by linkage threshold. A negative value of days between specimen and outcome date indicates the outcome occurring before the specimen date.

Days from specimen to outcome date	Count	Proportion of positive tests
Positive tests	3542	
Hospital spell admission		
[-28, 0]	1654	0.467
[1,14]	126	0.036
[15,28]	80	0.023
Hospital episode with norovirus diagnosis		
[-28, 0]	722	0.204
[1,14]	35	0.01
[15,28]	3	0.001
Death		
[0,14]	89	0.025
[15,28]	89	0.025
[29,60]	112	0.032
[61,100]	96	0.027

Appendix Table 2. Unadjusted raw counts and case-fatality ratios of de-duplicated positive norovirus test results from MOLIS-deaths data (MOLIS tests linked to deaths within 14 days). This is for all-cause deaths within 14 days of a positive norovirus test result.

Covariate	Positive tests	Deaths	Deaths / positive tests
Overall			
	3542	98	0.028
Genotype			
GII.4	1297	50	0.039
GII.17	1019	26	0.026
Other	858	6	0.007
Unknown	368	16	0.043
Age group			
0-4	864	3	0.003
5-19	183	2	0.011
20-49	355	2	0.006
50-74	708	19	0.027
75+	1431	71	0.050
Region			
East of England	332	10	0.030
London	48	0	0.000
Midlands	301	12	0.040
North East and Yorkshire	476	9	0.019
North West	1609	45	0.028
South East	82	1	0.012
South West	694	21	0.030

Appendix Table 3. Unadjusted raw counts and case-fatality ratios of de-duplicated positive test results from SGSS-deaths data within 14 days by age group, genotype and healthcare level. This is for all-cause deaths within 14 days of a positive norovirus test result.

Covariate	Positive tests	Deaths	Deaths / positive tests
Overall			
	32427	1256	0.039
Age group			
0-4	4149	9	0.002
5-19	1036	5	0.005
20-49	3311	14	0.004
50-74	6856	201	0.029
75+	17073	1027	0.060
Healthcare level			
Primary care	3329	17	0.005
Secondary care	23187	1051	0.045
Other	2182	49	0.022
Unknown	3729	139	0.037
Lab region			
East Midlands	1272	63	0.050
East of England	2488	127	0.051
London	3059	60	0.020
North East	2927	117	0.040
North West	4775	191	0.040
South East	4015	148	0.037
South West	5387	230	0.043
West Midlands	4575	193	0.042
Yorkshire and The Humber	3929	127	0.032

Appendix Table 4. Unadjusted raw counts and case-fatality ratios of de-duplicated positive test results from MOLIS-SGSS-deaths within 14 days by age group, genotype and healthcare level. This is for all-cause deaths within 14 days of a positive norovirus test result.

Covariate	Positive tests	Deaths	Deaths / positive tests
Overall			
	1900	51	0.027
Age group			
0-4	293	2	0.007
5-19	89	1	0.011
20-49	223	0	0.000
50-74	391	6	0.015
75+	904	42	0.046
Genotype			
GII.4	705	30	0.043
GII.17	583	12	0.021
Other	519	4	0.008
Unknown	93	5	0.054
Healthcare level			
Primary care	266	2	0.008
Secondary care	934	39	0.042
Other	566	8	0.014
Unknown	134	2	0.015
Lab broad region			
North	915	24	0.026
Midlands	141	5	0.035
South	844	22	0.026

Appendix Table 5. Case-fatality risk by genotype and time period, for all-cause death within 14 days of a positive norovirus test for an average person in the MOLIS-deaths cohort (median age = 64, modal region = North West). Time period facets were chosen to reflect periods of peak activity in each season. Two time periods are shown for the 2023/24 season to reflect periods of GII.4 or GII.17 dominance. In order, the time periods refer to test results with specimen date in the calendar quarter beginning January 2023, January 2024, April 2024 and January 2025.

Genotype	CFR % (95% CI)
2022/23 (GII.4 dominant)	
GII.4	4.59 (2.02, 10.55)
GII.17	2.57 (0.90, 7.35)
2023/24 (GII.4 dominant)	
GII.4	4.07 (1.71, 9.89)
GII.17	2.28 (0.86, 6.17)
2023/24 (GII.17 dominant)	
GII.4	3.33 (1.19, 9.55)
GII.17	1.86 (0.64, 5.56)
2024/25 (GII.17 dominant)	
GII.4	3.11 (1.29, 7.60)
GII.17	1.74 (0.65, 4.67)

Appendix Table 6. Case-fatality risk for all-cause death within 14 days of a positive norovirus test for an average case in the SGSS-deaths cohort (median age = 75, modal region = South West) identified in primary or secondary care. Time period facets were chosen to reflect periods of peak activity in each season. Two time periods are shown for the 2023/24 season to reflect periods of GII.4 or GII.17 dominance. In order, the time periods refer to test results with specimen date in the calendar quarter beginning January 2023, January 2024, April 2024 and January 2025.

Healthcare level	CFR % (95% CI)
2022/23 (GII.4 dominant)	
Primary care	0.45 (0.16, 1.23)
Secondary care	4.94 (3.92, 6.21)
2023/24 (GII.4 dominant)	
Primary care	0.41 (0.15, 1.14)
Secondary care	4.54 (3.54, 5.81)
2023/24 (GII.17 dominant)	
Primary care	0.39 (0.14, 1.09)
Secondary care	4.27 (3.10, 5.86)
2024/25 (GII.17 dominant)	
Primary care	0.42 (0.15, 1.16)
Secondary care	4.64 (3.68, 5.85)

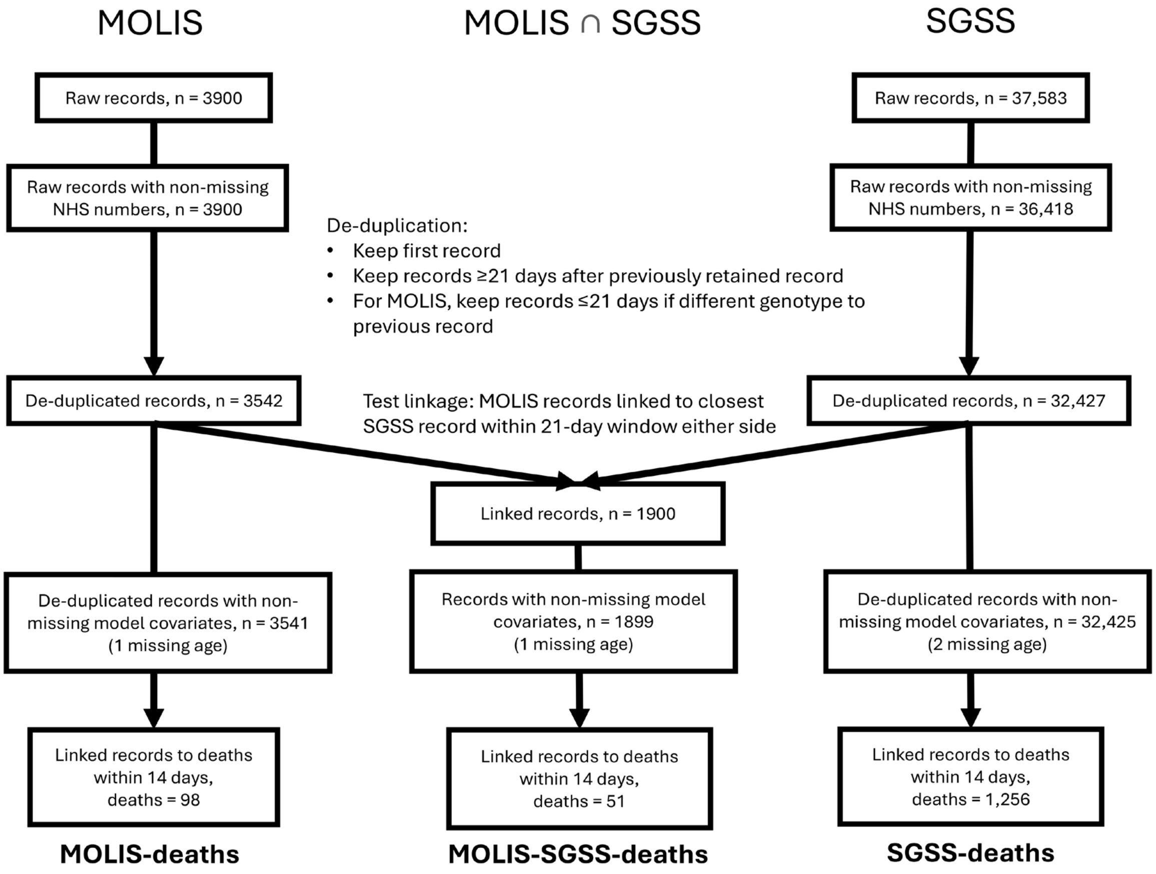

Appendix Figure 1. Data flow from raw data sources to de-duplicated linked test result records and deaths that are used in the case-fatality models.

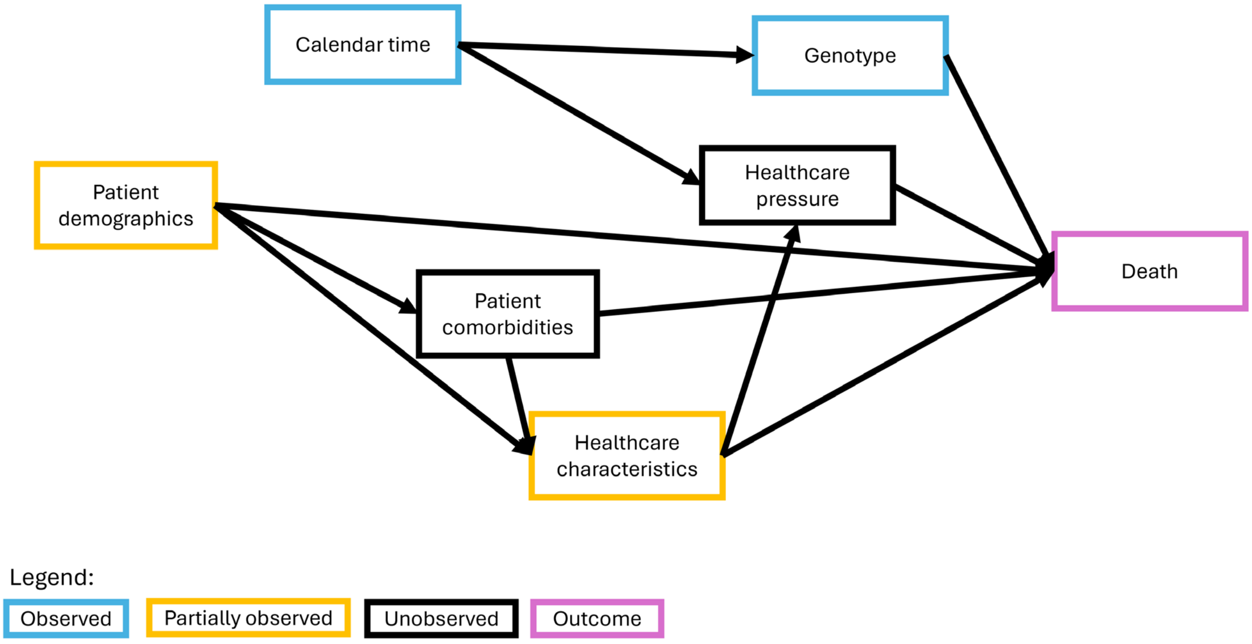

Appendix Figure 2. Directed acyclic graph (DAG) of causal effects contributing to the death of a detected norovirus case, whether attributable to norovirus or not. Observed effects are in blue, partially observed effects in orange, and the outcome (death) in purple. Arrows show the direction of causal relationships.

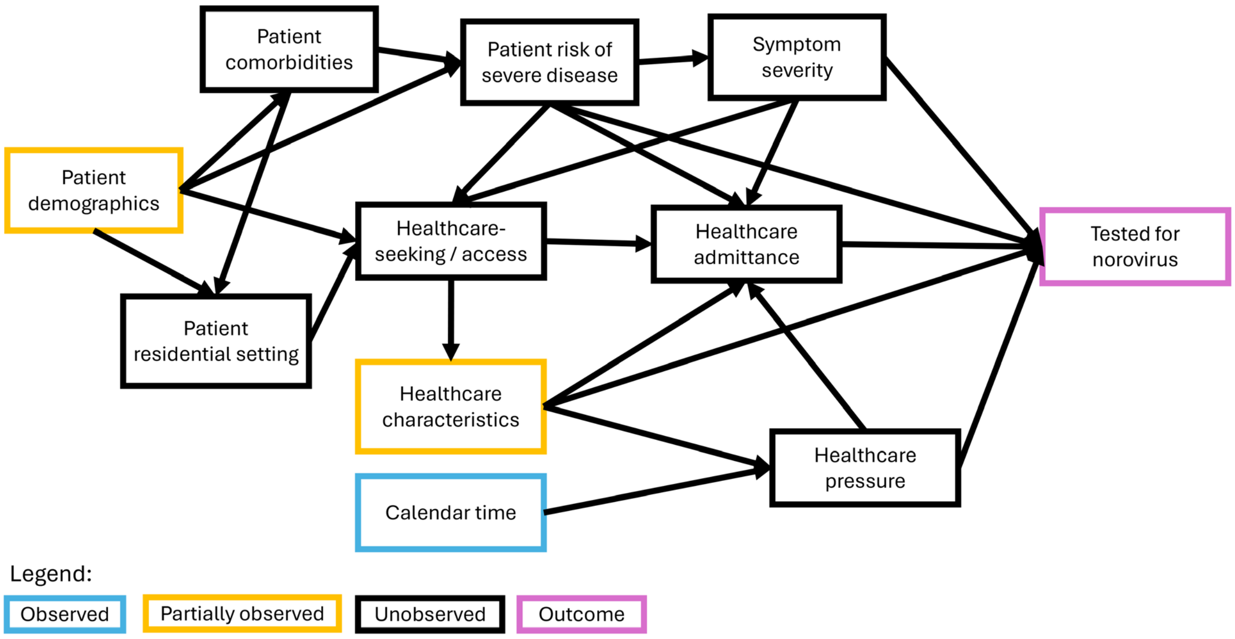

Appendix Figure 3. Directed acyclic graph (DAG) of causal effects contributing to an individual with norovirus being tested. Observed effects are in blue, partially observed effects in orange, and the outcome in purple. Arrows show the direction of causal relationships. This does not include the decision to genotype specimens, which may have additional factors.

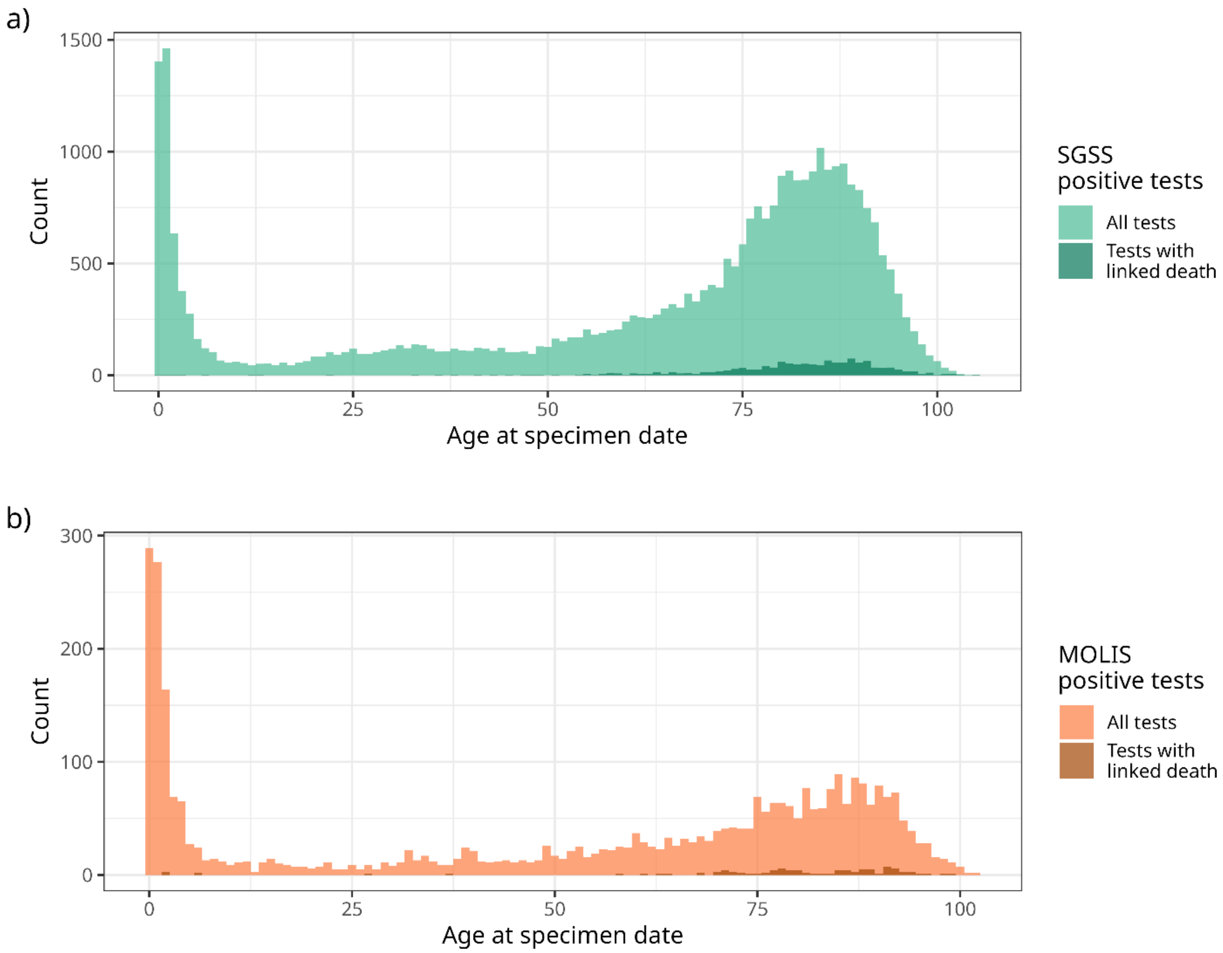

Appendix Figure 4. Age distributions of de-duplicated positive test results and linked deaths within 14 days for A) SGSS-deaths and B) MOLIS-deaths data.

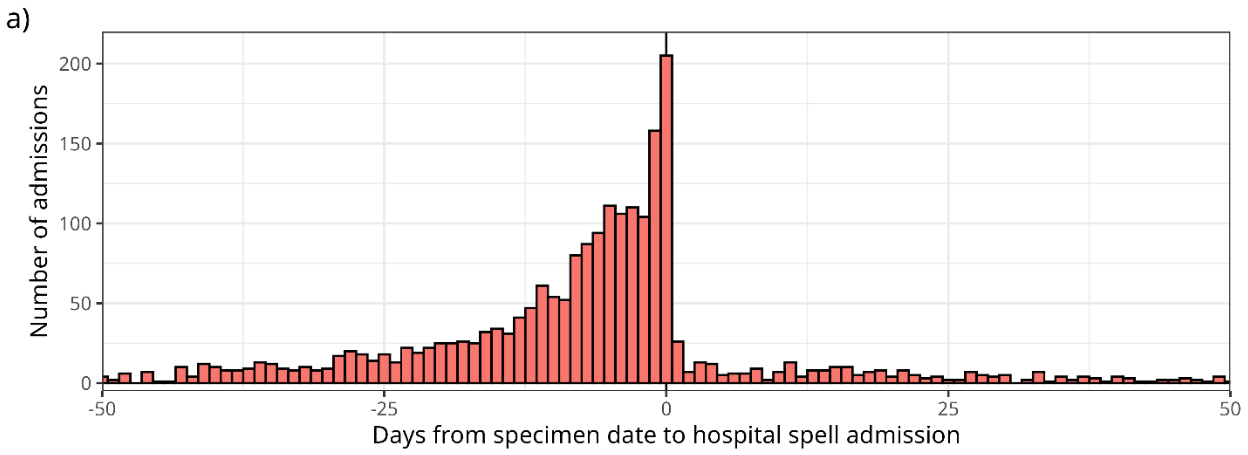

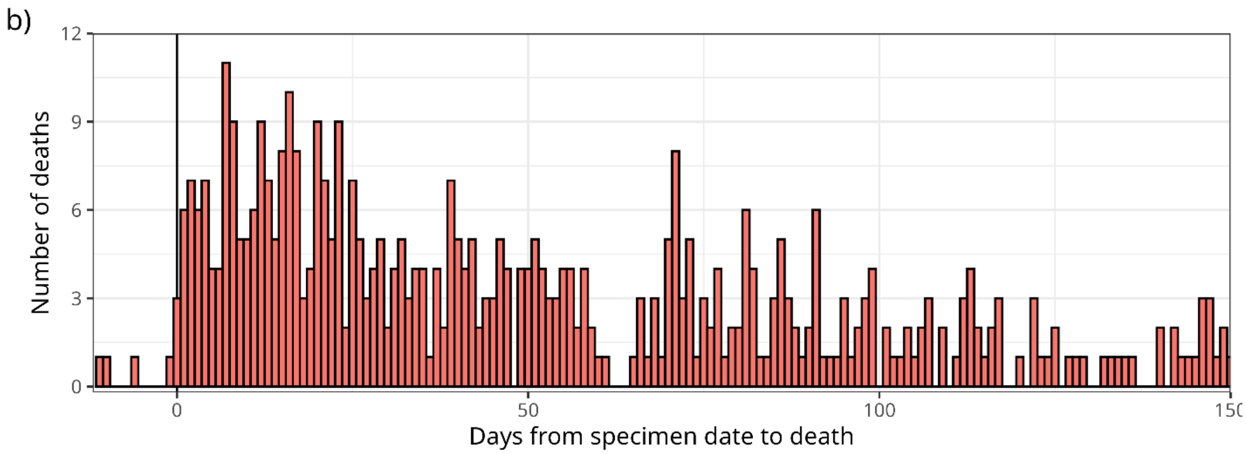

Appendix Figure 5. For outcomes linked to a positive norovirus test from MOLIS, time from specimen date to: A) hospital spell admission date (restricted to between -50 and 50 days), and B) death (up to 150 days). A negative value of days from specimen date to outcome indicates the outcome occurring before the specimen date.

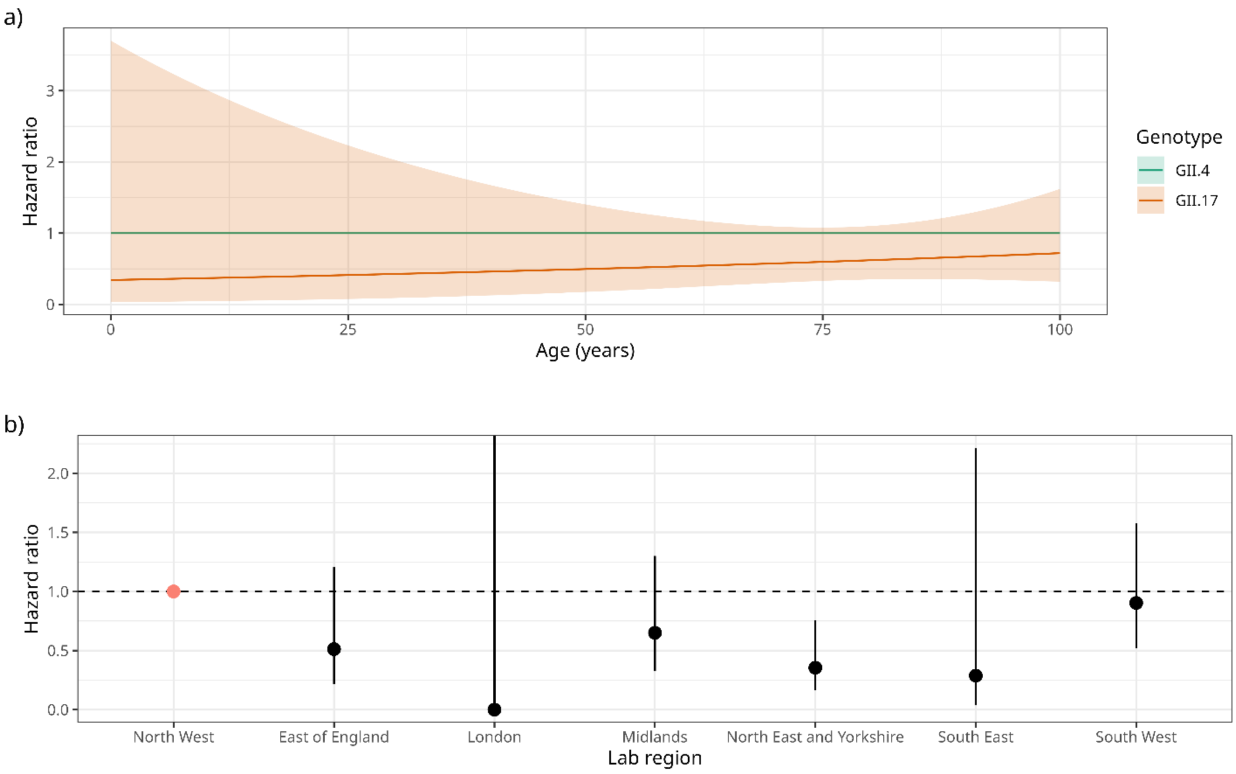

Appendix Figure 6. Hazard ratios and 95% CIs for genotype by A) age and B) region from CFR model using MOLIS-deaths data. Linkage threshold is death within 14 days. The most abundant categories were chosen as the reference genotype (GII.4) and region (North West).

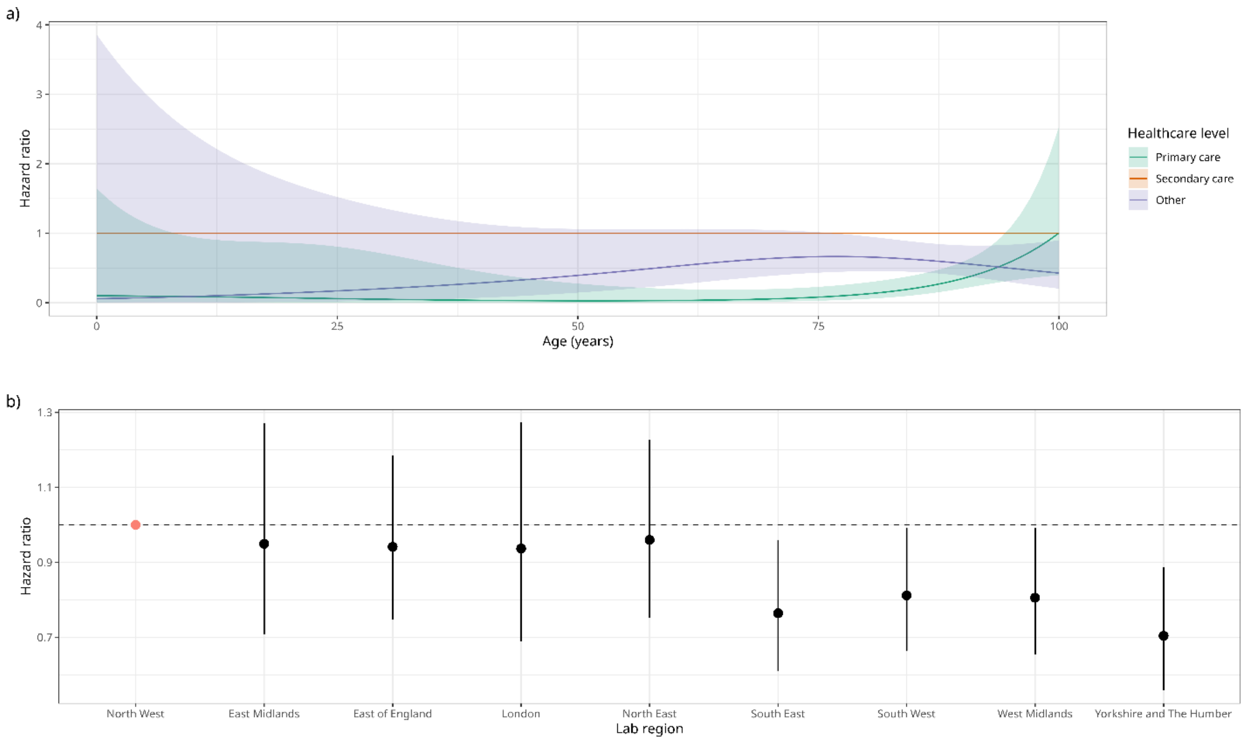

Appendix Figure 7. Hazard ratios and 95% CIs for healthcare level by A) age and B) lab region from CFR model using SGSS-deaths data. Linkage threshold is death within 14 days. The most abundant category was chosen as the reference healthcare level (Secondary care). The reference lab region (North West) was chosen to match the reference lab region in the MOLIS model.

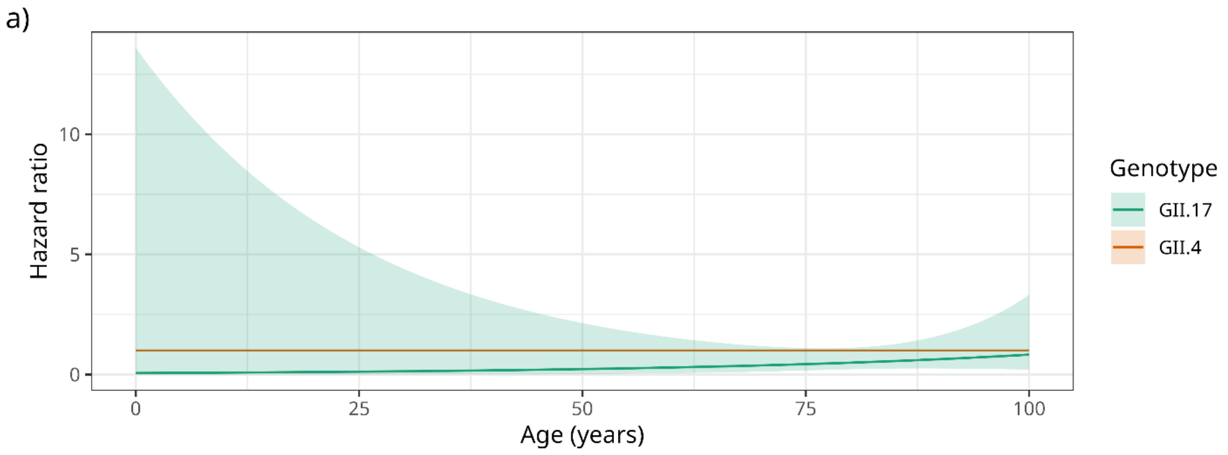

b)

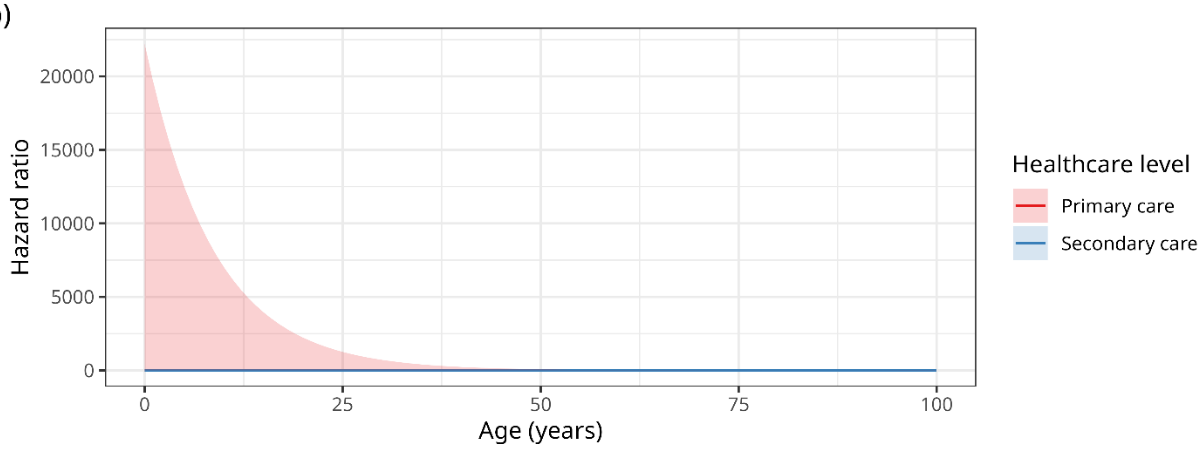

c)

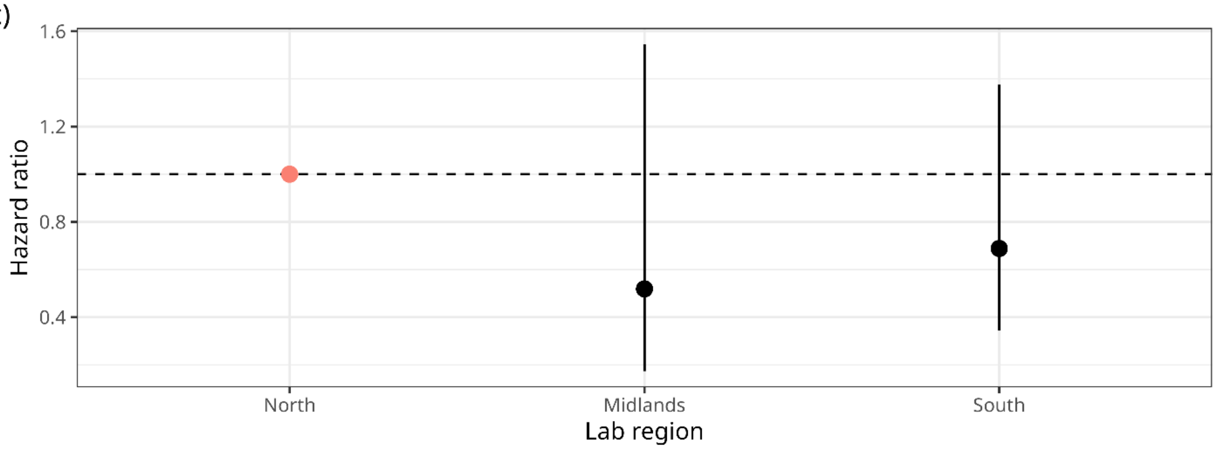

Appendix Figure 8. Hazard ratios and 95% CIs for genotype from CFR model using MOLIS-SGSS-deaths by A) genotype and age, B) healthcare level and age, and C) lab broad region. Linkage threshold is death within 14 days. The most abundant categories were chosen as the reference genotype (GII.4), healthcare level (Secondary care) and lab broad region (North).

a)

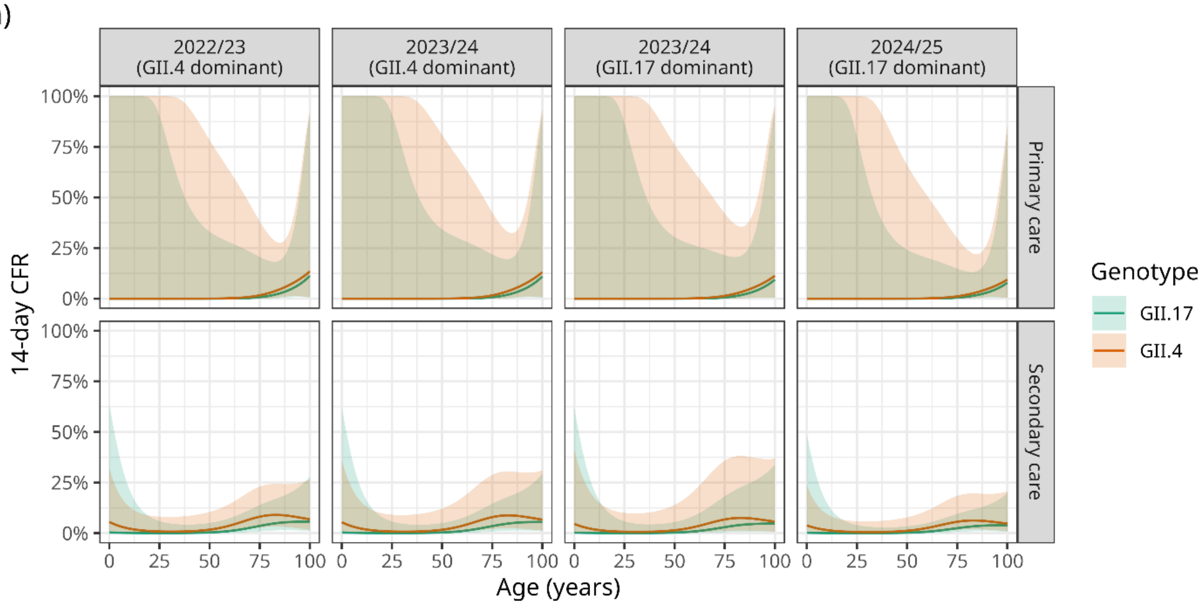

b)

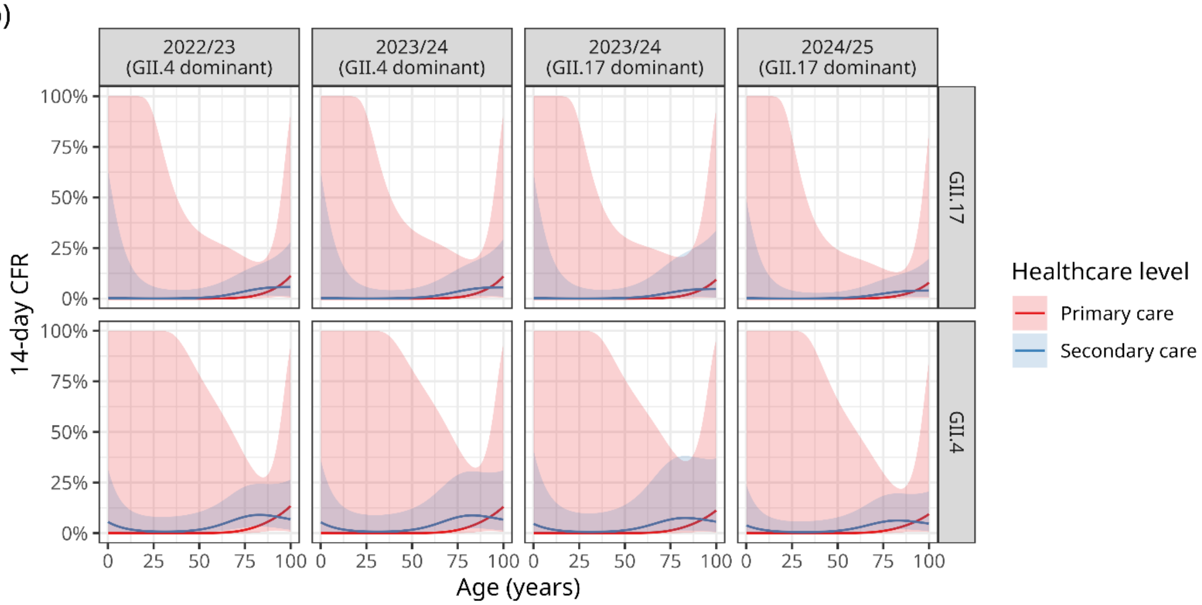

Appendix Figure 9. Predicted age-varying case fatality risk (CFR) for all-cause death within 14 days of a positive test with 95% confidence intervals by time period and A) genotype, and B) healthcare level using MOLIS-SGSS-deaths data. Time period facets were chosen to reflect periods of peak activity in each season. Two time periods are shown for the 2023/24 season to reflect periods of GII.4 or GII.17 dominance. In order, the time periods refer to test results with specimen date in the calendar quarter beginning January 2023, January 2024, April 2024 and January 2025.

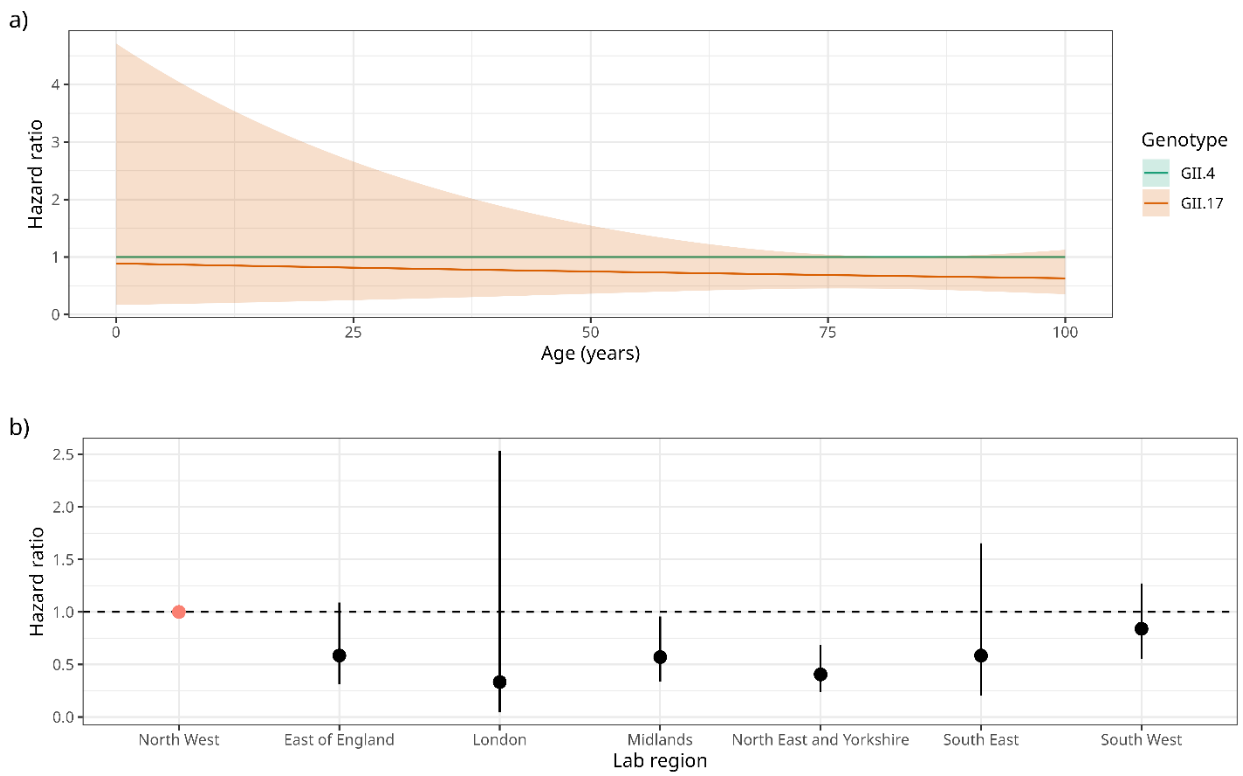

Appendix Figure 10. Hazard ratios and 95% CIs for A) genotype and B) lab region from CFR model using MOLIS-deaths data. Linkage threshold is death within 28 days. The most abundant categories were chosen as the reference genotype (GII.4) and lab broad region (North West).

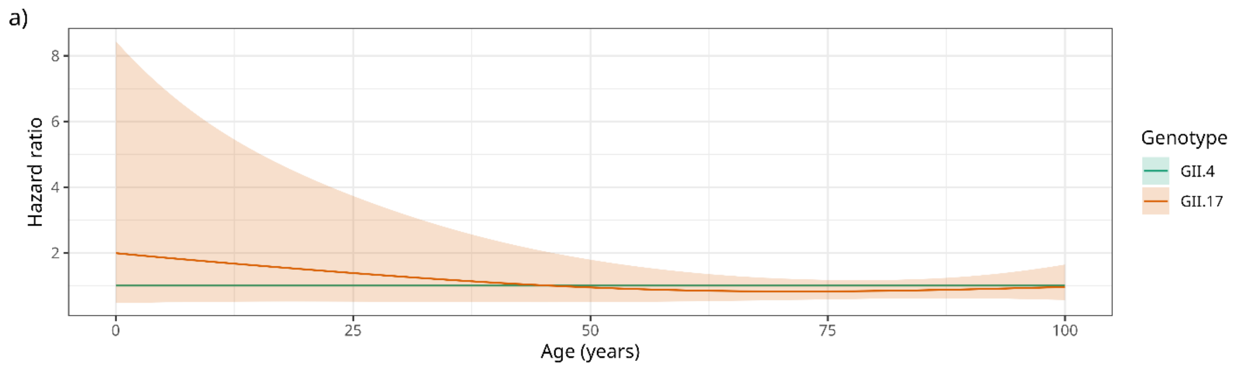

b)

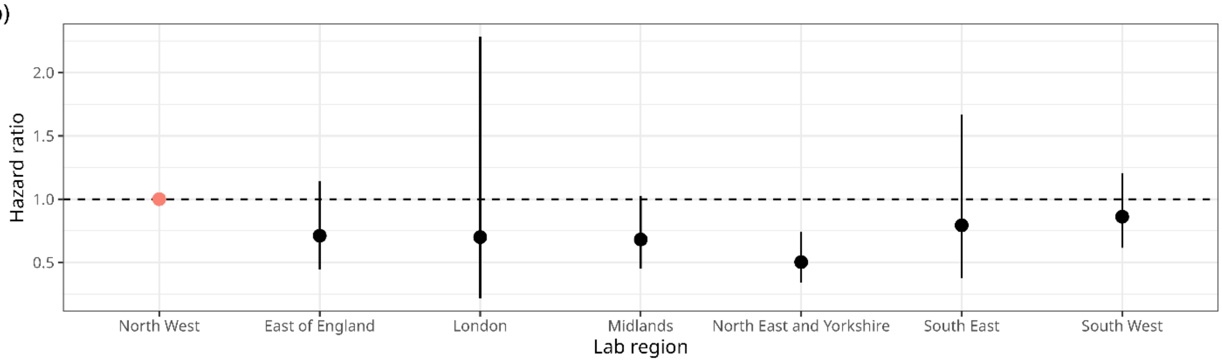

Appendix Figure 11. Hazard ratios and 95% CIs for A) genotype and B) lab region from CFR model using MOLIS-deaths data. Linkage threshold is death within 60 days. The most abundant categories were chosen as the reference genotype (GII.4) and lab broad region (North West).

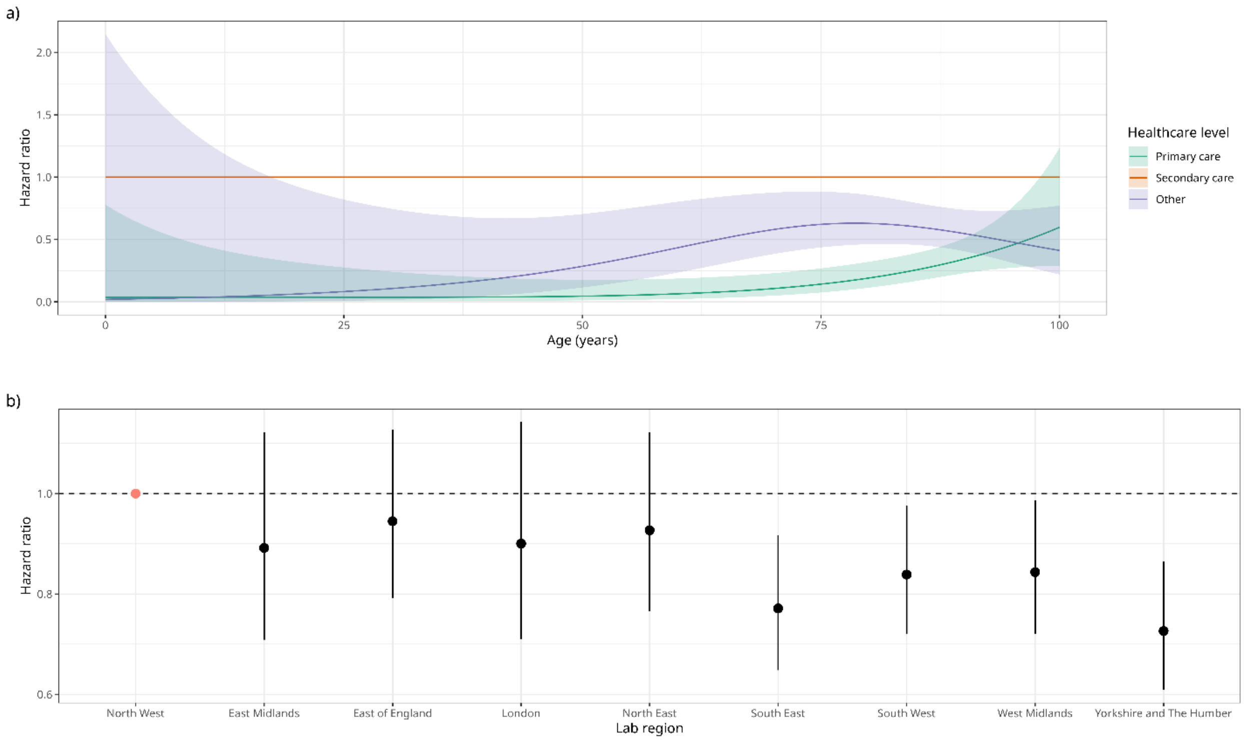

Appendix Figure 12. Hazard ratios and 95% CIs by A) healthcare level and age, and B) lab region from CFR model using SGSS-deaths data. Linkage threshold is death within 28 days. The most abundant category was chosen as the reference healthcare level (Secondary care). The reference lab region (North West) was chosen to match the reference lab region in the MOLIS model.

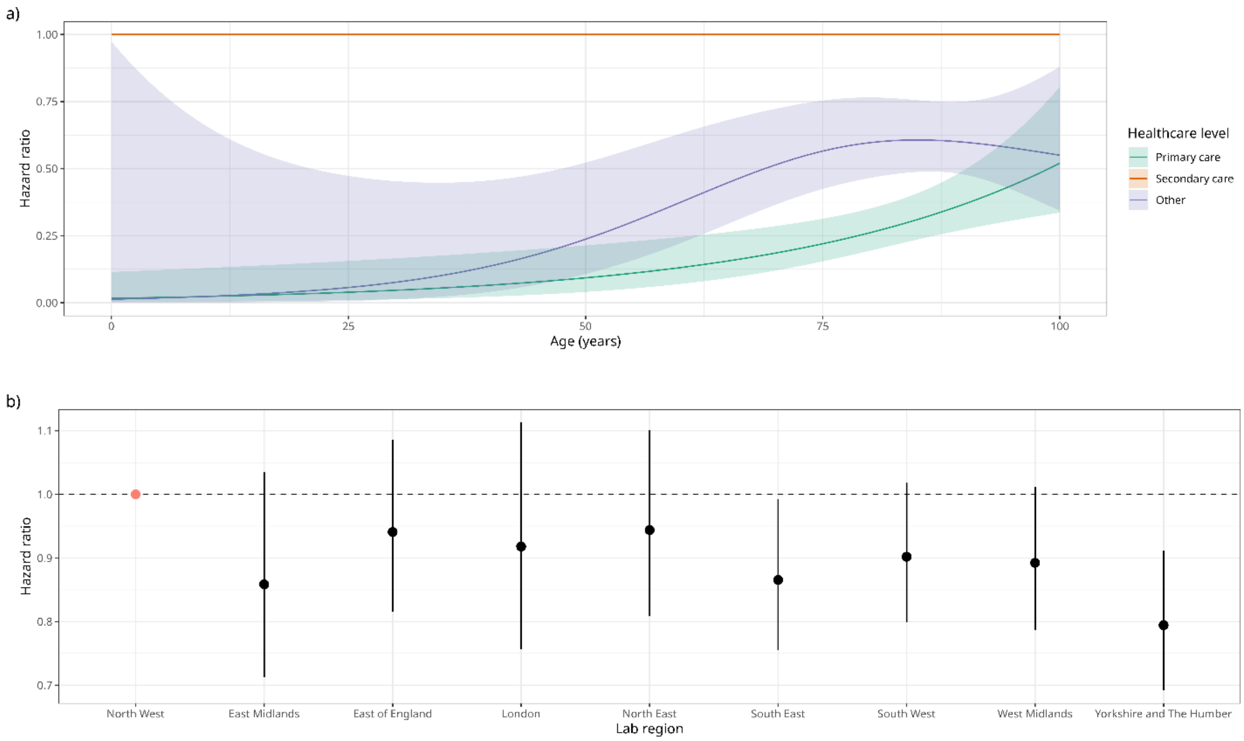

Appendix Figure 13. Hazard ratios and 95% CIs by A) healthcare level and age, and B) lab region from CFR model using SGSS-deaths data. Linkage threshold is death within 60 days. The most abundant category was chosen as the reference healthcare level (Secondary care). The reference lab region (North West) was chosen to match the reference lab region in the MOLIS model.

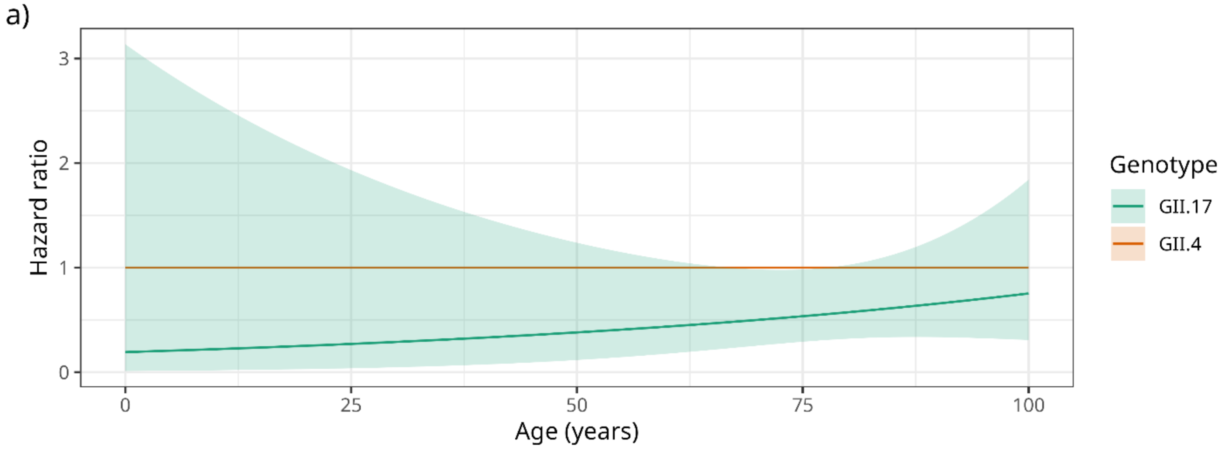

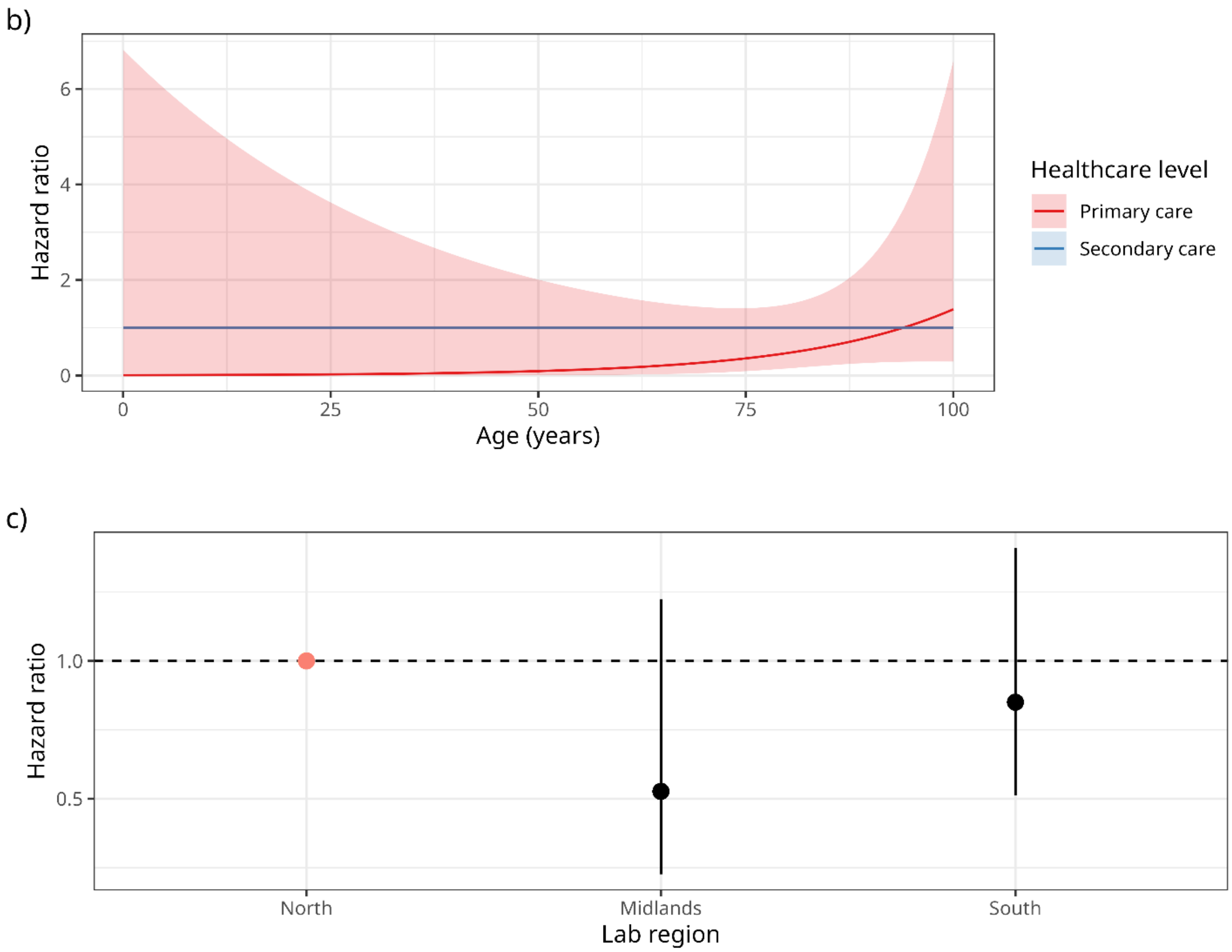

Appendix Figure 14. Hazard ratios and 95% CIs for A) genotype and age, B) healthcare level and age, and C) lab broad region from CFR model using MOLIS-SGSS-deaths data. Linkage threshold is death within 28 days. The most abundant categories were chosen as the reference genotype (GII.4), healthcare level (Secondary care) and lab broad region (North).

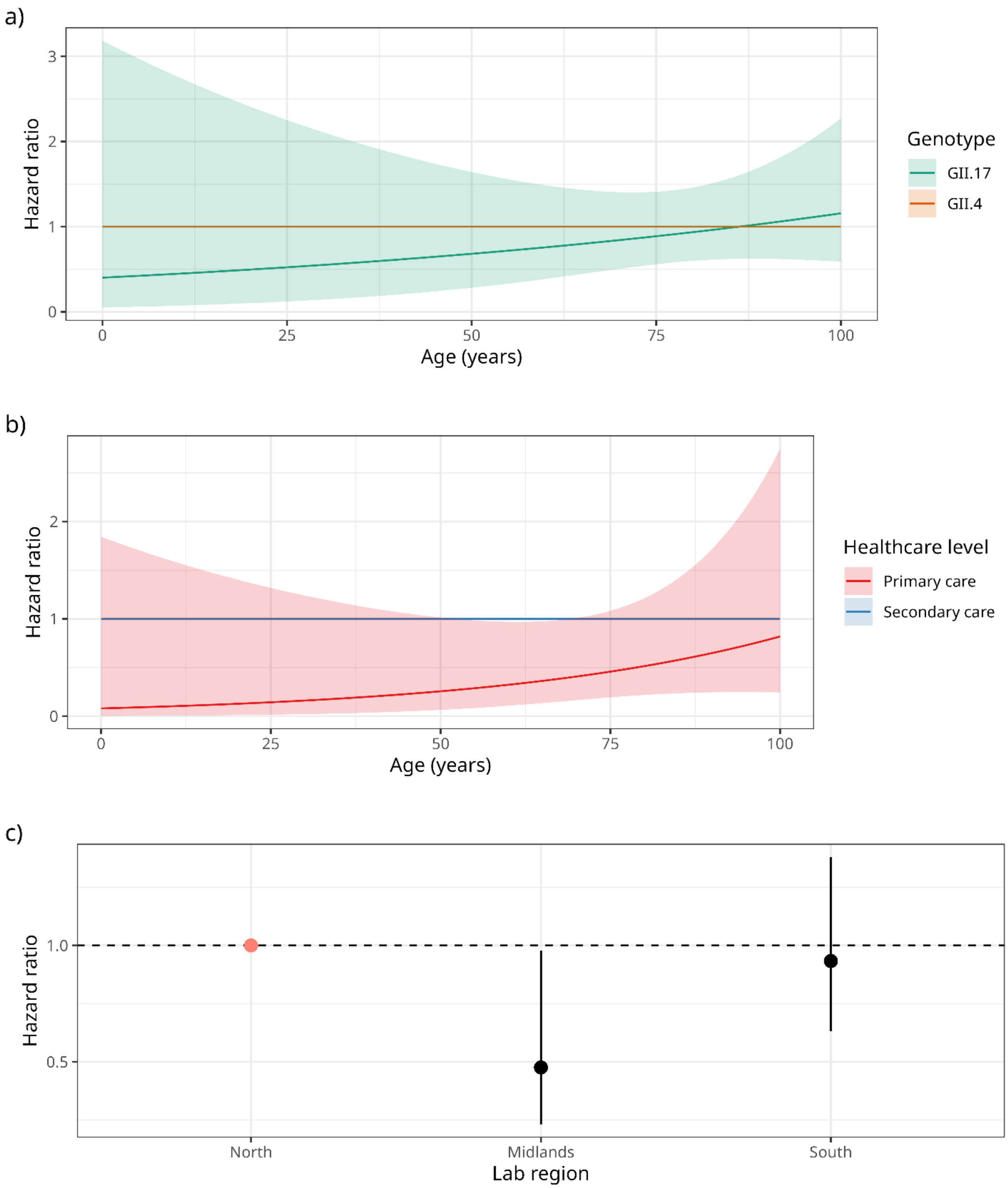

Appendix Figure 15. Hazard ratios and 95% CIs for A) genotype and age, B) healthcare level and age, and C) lab broad region from CFR model using MOLIS-SGSS-deaths data. Linkage threshold is death within 60 days. The most abundant categories were chosen as the reference genotype (GII.4), healthcare level (Secondary care) and lab broad region (North).
